# Phylogeography of the reef fish *Cephalopholis argus *(Epinephelidae) indicates Pleistocene isolation across the indo-pacific barrier with contemporary overlap in the coral triangle

**DOI:** 10.1186/1471-2148-11-189

**Published:** 2011-07-01

**Authors:** Michelle R Gaither, Brian W Bowen, Tiana-Rae Bordenave, Luiz A Rocha, Stephen J Newman, Juan A Gomez, Lynne van Herwerden, Matthew T Craig

**Affiliations:** 1 Hawaii Institute of Marine Biology University of Hawaii PO Box 1346, Kaneohe, HI 96744, USA; 2 Department of Ichthyology California Academy of Sciences 55 Music Concourse Drive San Francisco, CA 94118, USA; 3 Western Australian Fisheries and Marine Research Laboratories Department of Fisheries Government of Western Australia P.O. Box 20, North Beach, WA 6920, Australia; 4 School of Marine & Tropical Biology James Cook University Townsville, QLD 4811, Australia; 5 Department of Marine Sciences University of Puerto Rico Mayagüez P.O. Box 9000, Mayagüez PR 00681, USA

## Abstract

**Background:**

The Coral Triangle (CT), bounded by the Philippines, the Malay Peninsula, and New Guinea, is the epicenter of marine biodiversity. Hypotheses that explain the source of this rich biodiversity include 1) the center of origin, 2) the center of accumulation, and 3) the region of overlap. Here we contribute to the debate with a phylogeographic survey of a widely distributed reef fish, the Peacock Grouper (*Cephalopholis argus*; Epinephelidae) at 21 locations (N = 550) using DNA sequence data from mtDNA cytochrome *b *and two nuclear introns (gonadotropin-releasing hormone and S7 ribosomal protein).

**Results:**

Population structure was significant (Φ_ST _= 0.297, *P *< 0.001; *F*_ST _= 0.078, *P *< 0.001; *F*_ST _= 0.099, *P *< 0.001 for the three loci, respectively) among five regions: French Polynesia, the central-west Pacific (Line Islands to northeastern Australia), Indo-Pacific boundary (Bali and Rowley Shoals), eastern Indian Ocean (Cocos/Keeling and Christmas Island), and western Indian Ocean (Diego Garcia, Oman, and Seychelles). A strong signal of isolation by distance was detected in both mtDNA (r = 0.749, *P *= 0.001) and the combined nuclear loci (r = 0.715, *P *< 0.001). We detected evidence of population expansion with migration toward the CT. Two clusters of haplotypes were detected in the mtDNA data (*d *= 0.008), corresponding to the Pacific and Indian Oceans, with a low level of introgression observed outside a mixing zone at the Pacific-Indian boundary.

**Conclusions:**

We conclude that the Indo-Pacific Barrier, operating during low sea level associated with glaciation, defines the primary phylogeographic pattern in this species. These data support a scenario of isolation on the scale of 10^5 ^year glacial cycles, followed by population expansion toward the CT, and overlap of divergent lineages at the Pacific-Indian boundary. This pattern of isolation, divergence, and subsequent overlap likely contributes to species richness at the adjacent CT and is consistent with the region of overlap hypothesis.

## Background

Current efforts to identify and preserve biodiversity are dependent upon our ability to locate hotspots and to understand how that diversity is generated. Conservation efforts must preserve not just standing biodiversity but also the mechanisms that produce it [1]. The Coral Triangle (CT), bounded by the Philippines, the Malay Peninsula, and New Guinea, is the epicenter of marine biodiversity. Species diversity declines with distance from this region, both latitudinally and longitudinally, a pattern that applies to a broad array of taxa [2-8]. The generality of this pattern has led many to conclude that a common mechanism may be responsible for generating diversity in the CT. A number of hypotheses have been proposed to explain the source of the incredible number of species found in this region and these can be grouped into three categories: 1) center of origin, 2) center of accumulation, and 3) region of overlap.

The center of origin hypothesis was proposed by Ekman [9], who suggested that the CT is the primary source of biodiversity in the Indo-Pacific due to an unusually high rate of speciation in the region. He suggested that the decline in species richness with distance from the CT is an artifact of prevailing currents that impede outward dispersal [9]. The most common mechanism invoked to explain the proposed elevated speciation rate is the fracturing of populations as a result of the geological complexity of the region and eustatic sea level changes [10]. Others have suggested that increased rates of sympatric or parapatric speciation driven by different selection pressures in a heterogeneous environment could be contributing to the species richness of the CT [11,12]. Evidence for this argument includes the finding of fine scale population subdivisions within the CT [13-18].

In contrast, the center of accumulation hypothesis [19] proposes speciation in isolated peripheral locations with subsequent dispersal of novel taxa into the CT. The long history of the Pacific archipelagos, some of which date to the Cretaceous, and ocean current and wind patterns that favor dispersal toward the CT have been offered as a mechanism [19,20]. Finally, the region of overlap hypothesis [21] maintains that the high species diversity in the CT is due to the overlap of faunas from two biogeographic provinces: Indo-Polynesian and Western Indian Ocean [22]. The region roughly dividing these two provinces is west of the shallow Sunda and Sahul shelves of the East Indies. During the Pleistocene, sea level was as much as 130 m below present levels and produced a near continuous land bridge between Asia and Australia [23], greatly restricting dispersal between ocean basins in the region known as the Indo-Pacific Barrier (IPB). Isolation of conspecific populations across the IPB may have led to allopatric speciation and contributed to the distinction of the Pacific and Indian Ocean faunas. According to the region of overlap hypothesis, relaxation of the IPB following each Pleistocene glacial maximum has resulted in dispersal pathways between the Pacific and Indian Oceans with the CT representing the area of overlap between the two distinct biotas. The differences between the center of accumulation and region of overlap hypotheses are subtle. In both cases speciation occurs outside the CT with subsequent dispersal toward the CT. However, the region of overlap hypothesis is based on the premise that the isolating mechanism is the IPB with the faunas of the Pacific and Indian Oceans diverging during periods of restricted dispersal. In contrast, the center of accumulation hypothesis does not specify a mechanism of divergence nor is it associated with any biogeographic barrier. This hypothesis invokes speciation in peripheral locations, followed by dispersal to the CT on prevailing oceanic currents.

Contemporary species distributions are the most common line of evidence offered to examine these hypotheses yet no consensus has evolved. Mora et al. [7] examined the ranges of nearly 2,000 Indo-Pacific fishes and found that the midpoint of their ranges centered on the CT, a result they interpret as evidence for the center of origin hypothesis. Connolly et al. [24], using a midpoint domain model, found evidence for the accumulation of taxa in the CT due to species dispersing on oceanic gyres. Halas and Winterbottom [25] employed a novel phylogenetic approach to address the issue but found no conclusive evidence for any of the hypotheses. Evidence for a combined influence of all these processes in generating the high biodiversity in the CT has led many to conclude that the processes are not mutually exclusive and act simultaneously [3,26-28].

Patterns of genetic variation in widely distributed species, while not often employed to address the source of biodiversity hotspots, provide a historical perspective that cannot be resolved with contemporary species distributions. Each hypothesis results in specific predictions about geographic positioning of new species and lineages within species [29]. The center of origin hypothesis predicts that the oldest populations (within new species) will be in the CT, possibly with decreasing haplotype diversity emanating from the center similar to the observed decline in species richness (sensu [30]). In contrast, the center of accumulation hypothesis predicts that the oldest populations (within new species) will be found peripheral to the CT accompanied with unidirectional dispersal toward the CT. Similar to the center of origin, the region of overlap hypothesis predicts that the most diverse (but not oldest) populations will be centered in the CT, however in this case the high diversity is the result of the overlap of divergent lineages from peripheral regions. While there have been a handful of intraspecific genetic studies that address the origin of diversity in the CT, the results are conflicting. Evidence for the center of accumulation hypothesis has been found in the Lemon Damselfish (*Pomacentrus moluccensis*) [29] and the Yellow Tang (*Zebrasoma flavescens*) [31]. On the other hand, sea urchins [32] and wrasses [33] invoke a combination of the center of origin and the center of accumulation hypotheses. Of course all of these conclusions, including our own, are premised on the assumption that intraspecific genetic divergences translate into macroevolutionary (interspecific) partitions [34].

**Figure 1:**
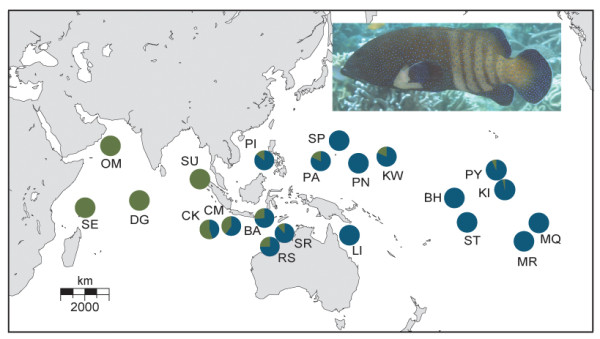
**Map of study area**. Pie charts represent the ratio of individuals at each location with either the Pacific or Indian Ocean lineage as defined in Figure 2 (Photo credit: Luiz Rocha).

Here we contribute a range-wide phylogeographic study of a widely distributed grouper to test competing hypotheses concerning the origins of biodiversity in the CT. The Peacock Grouper, *Cephalopholis argus *(Bloch and Schneider 1801), is a demersal (bottom dwelling) reef fish of the family Epinephelidae. This species is found in reef habitat (2-40 m depth) from the Pitcairn group in the Pacific to east Africa and the Red Sea [[35], Figure 1]. Many members of the genus *Cephalopholis *display complex social behaviors such as territoriality, sequential hermaphroditism, and a haremic social system [36]. Long-range dispersal in this species, as in most coral reef organisms, is limited to the pelagic larval stage [37]. The pelagic larval duration for *C. argus *has not been determined but a 40-day average is proposed for Epinephelids [38]. We analyzed DNA sequence data to assess phylogeographic patterns across the range of this species to test three alternative hypotheses concerning the origin of the biodiversity in the CT. Explicitly we address the following questions: 1) does genetic diversity in the CT indicate an ancestral population with dispersal away from the CT as would be expected under the center of origin hypothesis, 2) is the ancestral diversity peripheral to the CT and accompanied with evidence of migration toward the CT as would be expected under the center of accumulation hypothesis, or 3) is the genetic diversity in the CT the result of mixing of divergent lineages across the IPB as would be expected under the region of overlap hypothesis?

## Methods

A total of 550 *Cephalopholis argus *were collected from 21 locations across the species range in the Pacific and Indian Oceans including two locations at opposite ends of the CT (Philippines and Bali; Table 1). Most samples were collected by SCUBA divers using polespears or by fishers using lines. In some cases, samples were obtained from fish markets but only when we were confident they had been caught locally (within 100 km). Tissues samples (fin clips or gill filaments) were preserved in salt-saturated DMSO [39] and stored at room temperature. DNA was isolated using the modified HotSHOT method [40,41]. Approximately 870 bp of mitochondrial cytochrome *b *(Cyt*b*) were amplified using the primers CB6F (5'-CTCCCTGCACCTTCAAACAT-3') and CB6R (5'-GGAAGG TTAAAG CCC GTTGT-3') which we designed for this species. Additionally, approximately 375 bp of the third intron in the gonadotropin-releasing hormone (GnRH) gene were amplified using the primers GnRH3F and GnRH3R [42] and approximately 730 bp of the first intron of the S7 ribosomal protein (S7) gene were amplified using the primers S7RPEX1F and S7RPEX2R [43].

**Table 1:** Molecular diversity indices for 21 populations of *Cephalopholis argus *

	*Cytb*				*GnRH*					*S7*				
***Sample Location***	***N***	***N*_h_**	***h***	***π***	***N***	***N*_a_**	***H*_O_**	***H*_E_**	***P*-value**	***N***	***N*_a_**	***H*_O_**	***H*_E_**	***P*-value**

Marquesas (MQ)	50	8	0.65 ± 0.06	0.002 ± 0.001	34	3	0.21	0.44	0.002	48	9	0.52	0.60	0.596
Moorea (MR)	36	5	0.38 ± 0.10	0.001 ± 0.001	33	2	0.24	0.26	0.549	34	7	0.59	0.60	0.298
Kiritimati (KI)	32	8	0.74 ± 0.05	0.003 ± 0.002	33	3	0.55	0.49	0.335	28	7	0.36	0.40	0.388
Palmyra (PY)	29	6	0.65 ± 0.07	0.002 ± 0.002	30	3	0.30	0.33	0.217	27	6	0.33	0.33	0.337
Samoa/Tokelau (ST)	27	6	0.64 ± 0.07	0.001 ± 0.001	21	4	0.29	0.33	0.044	24	6	0.25	0.34	0.076
Baker/Howland (BH)	27	6	0.68 ± 0.06	0.002 ± 0.001	27	4	0.52	0.55	0.767	27	7	0.37	0.39	0.365
Kwajalein (KW)	22	10	0.86 ± 0.05	0.005 ± 0.003	23	5	0.48	0.52	0.846	23	7	0.26	0.48	< 0.001
Pohnpei (PN)	15	6	0.74 ± 0.09	0.004 ± 0.002	15	3	0.20	0.38	0.055	15	8	0.67	0.67	0.709
Saipan (SP)	19	6	0.77 ± 0.07	0.003 ± 0.002	19	3	0.37	0.56	0.570	18	6	0.56	0.50	0.271
Palau (PA)	22	8	0.77 ± 0.07	0.004 ± 0.002	23	5	0.57	0.47	0.897	23	6	0.52	0.60	0.128
Lizard Island (LI)	12	5	0.67 ± 0.14	0.001 ± 0.001	10	3	0.40	0.35	1.000	7	3	0.29	0.28	1.000
Philippines (PI)	6	4	0.87 ± 0.13	0.009 ± 0.006	5	3	0.60	0.71	1.000	5	4	0.60	0.64	0.644
Bali (BA)	23	7	0.81 ± 0.05	0.005 ± 0.003	19	4	0.47	0.60	0.054	16	7	0.69	0.76	0.322
Scott Reef (SR)	42	8	0.73 ± 0.05	0.004 ± 0.002	41	7	0.56	0.58	0.262	39	8	0.72	0.64	0.897
Rowley Shoals (RS)	40	9	0.81 ± 0.04	0.005 ± 0.003	33	7	0.67	0.65	0.073	30	10	0.80	0.77	0.780
Christmas Island (CM)	49	10	0.83 ± 0.02	0.006 ± 0.004	47	7	0.55	0.57	0.262	49	11	0.71	0.70	0.717
Cocos/Keeling (CK)	40	9	0.87 ± 0.02	0.006 ± 0.004	30	10	0.83	0.78	0.161	29	7	0.72	0.71	0.599
Sumatra (SU)	4	3	0.83 ± 0.22	0.007 ± 0.005	6	5	0.83	0.82	0.703	5	4	0.60	0.71	1.000
Diego Garcia (DG)	33	10	0.87 ± 0.03	0.003 ± 0.002	33	8	0.82	0.80	0.295	33	9	0.76	0.66	0.047
Oman (OM)	9	5	0.81 ± 0.12	0.006 ± 0.003	4	4	0.75	0.64	1.000	7	6	0.71	0.79	0.869
Seychelles (SE)	13	10	0.96 ± 0.04	0.003 ± 0.002	13	5	0.62	0.80	0.054	13	5	0.77	0.70	0.739

All samples	550	55	0.80 ± 0.01	0.005 ± 0.003	499	11		0.58		500	20		0.67	

Sample locations and number of individuals (*N*) are shown. Number of haplotypes (*N*_h_), haplotype diversity (*h*), and nucleotide diversity (*π*) are listed for the cytochrome *b *dataset. Number of alleles (*N*_a_), observed heterozygosity (*H*_O_), and expected heterozygosity (*H*_E_) are listed for the gonadotropin releasing hormone (GnRH), and S7 ribosomal protein gene (S7) nuclear introns. *P*-values are the result of exact tests for Hardy-Weinberg equilibrium using a Markov chain with 100,000 steps in ARLEQUIN 3.5 [42].

Polymerase chain reactions (PCRs) for all three markers were carried out in a 10 μl volume containing 2-15 ng of template DNA, 0.2-0.3 μM of each primer, 5 μl of the premixed PCR solution BioMix Red™(Bioline Inc., Springfield, NJ, USA), and deionized water to volume. PCR reactions utilized the following cycling parameters: initial denaturation at 95°C and final extension at 72°C (10 min each), with an intervening 35 cycles of 30 s at 94°C, 30 s at the annealing temperature (54°C for Cyt*b*; 58°C for GnRH and S7), and 45 s at 72°C. Amplification products were purified using 0.75 units of Exonuclease I/0.5 units of Shrimp Alkaline Phosphatase (ExoSAP; USB, Cleveland, OH, USA) per 7.5 μl PCR products at 37°C for 60 min, followed by deactivation at 80°C for 10 min. DNA sequencing was performed with fluorescently-labeled dideoxy terminators on an ABI 3730XL Genetic Analyzer (Applied Biosystems, Foster City, CA, USA) at the University of Hawaii's Advanced Studies of Genomics, Proteomics, and Bioinformatics sequencing facility.

Sequences for each locus were aligned, edited, and trimmed to a common length using the DNA sequence assembly and analysis software GENEIOUS PRO 5.0 (Biomatters, LTD, Auckland, NZ). In all cases, alignment was unambiguous with no indels or frameshift mutations. Allelic states of nuclear sequences with more than one heterozygous site (GnRH = 43.1% and S7 = 48.4% of individuals) were estimated using the Bayesian program PHASE 2.1 [44,45] as implemented in the software DnaSP 5.0 [46]. We conducted six runs in PHASE for each dataset. Each run had a unique random-number seed. Five runs were conducted for 1000 iterations with 1000 burn-in iterations. To ensure proper allele assignment, a sixth run of 10000 iterations was conducted. All runs returned consistent allele identities. GnRH and S7 genotyptes resulted in no more than 4 and 6 ambiguous sites per individual, respectively. PHASE was able to differentiate all alleles with > 95% probability at both loci except at single nucleotide positions in 4 individuals at GnRH and 10 individuals at S7 or 0.8% and 2.0% of samples, respectively. Unique haplotypes and alleles were identified with the merge taxa option in MacClade 4.05 [47] and deposited in GenBank [ascension numbers: JN157683-JN157739 (Cyt*b*), JN157740-JN157750 (GnRH intron), JN157663-JN157682 (S7 intron)].

### Data analyses

#### Mitochondrial DNA

Summary statistics for *C. argus*, including haplotype diversity (*h*) and nucleotide diversity (π), were estimated with algorithms from Nei [48] as implemented in the statistical software package ARLEQUIN 3.5 [49]. To test whether haplotype and nucleotide diversities differed between ocean basins (Pacific Ocean = Marquesas, Moorea, Kiritimati, Palmyra, Samoa/Tokelau, Baker/Howland, Kwajalein, Pohnpei, Saipan, Palau, Lizard Island, and Philippines; Indian Ocean = Sumatra, Bali, Scott Reef, Rowley Shoals, Christmas Island, Cocos/Keeling, Diego Garcia, Oman, and Seychelles) we calculated unpaired *t*-tests using the online calculator GraphPad (http://www.graphpad.com/quickcalcs/ttest1.cfm). The AIC implemented in jMODELTEST 0.1.1 indicated the TPM1uf+G as the best-fit model of DNA sequence evolution with a gamma value of 0.065. Median-joining networks were constructed using the program NETWORK 4.5 with default settings [50]. An intra-specific phylogeny was produced using maximum likelihood (ML) methods and default settings in the program RAXML 7.2.7 [51]. Trees were rooted using Cyt*b *sequences of two congenerics (*C. urodeta *and *C. taeniops*) obtained from GenBank (ascension numbers AY786426 and EF455990, respectively). Bootstrap support values were calculated using default settings with 1000 replicates. The ML tree topology was confirmed by neighbor-joining (NJ) and Bayesian Markov Chain Monte Carlo (MCMC) analysis using MEGA 4.0 [52] and MRBAYES 3.1.1 [53], respectively. The NJ tree was generated using the Tamura-Nei model of evolution [54] and a gamma parameter of 0.065. Bootstrap support values were calculated using 1000 replicates. The Bayesian analysis was run using the default four heated, one million step chains with an initial burn-in of 100,000 steps. We calculated the corrected average number of pairwise differences between mitochondrial lineages (*d*) in ARLEQUIN.

To determine whether the number of pairwise differences among all DNA sequences reflected expanding or stable populations [55], we calculated the frequency distribution of the number of mutational differences between haplotypes (mismatch analyses), as implemented in ARLEQUIN. To determine confidence intervals around this value we calculated Harpending's raggedness index, r [55], which tests the null hypothesis of an expanding population. This statistic quantifies the smoothness of the observed pairwise difference distribution and a non-significant result indicates an expanding population. Fu's *F*_S _[56], which is highly sensitive to population expansions was calculated using 10,000 permutations. Significant negative values of *F*_S _indicate an excess of low-frequency haplotypes, a signature characteristic of either selection or a recent demographic expansion [56].

To test for hierarchical population genetic structure in *C. argus*, an analysis of molecular variance (AMOVA) was performed in ARLEQUIN using 20,000 permutations. Because the TPM1uf+G model of sequence evolution is not implemented in ARLEQUIN, we used the most similar model available [54] with a gamma value of 0.065. An analogue of Wright's *F*_ST _(Φ_ST_), which incorporates the model of sequence evolution, was calculated for the entire dataset and for pairwise comparisons among all locations. We maintained α = 0.05 among all pairwise tests by controlling for the false discovery rate as recommended by Benjamini and Yekutieli [57] and reviewed by Narum [58]. A Mantel test was performed to determine whether significant isolation-by-distance exists among populations by testing for correlation between pairwise Φ_ST _values and geographic distance using the Isolation-by-Distance Web Service 3.16 [59]. Mantel tests were performed with 10,000 iterations on the dataset that included negative Φ_ST _values and again with negative Φ_ST _values converted to zeros.

To estimate the time to coalescence we used the Bayesian MCMC approach implemented in BEAST 1.5.4 [60]. We conducted our analysis with a relaxed lognormal clock and under a model of uncorrelated substitution rates among branches. We used default priors under the HKY + G model of mutation (jMODELTEST) [61,62] and ran simulations for 10 million generations with sampling every 1000 generations. Five independent runs were computed to ensure convergence and log files were combined using the program TRACER 1.5 [63].

#### Nuclear introns

Observed heterozygosity (*H*_O_) and expected heterozygosity (*H*_E_) were calculated for each locus and an exact test of Hardy-Weinberg equilibrium (HWE) using 100,000 steps in a Markov chain was performed using ARLEQUIN. To test whether *H*_E _differed between ocean basins we calculated unpaired t-tests as described above. Linkage disequilibrium between the two nuclear loci was assessed using the likelihood ratio test with 20,000 permutations in ARLEQUIN. We tested for population expansions by calculating Fu's *F*_S _[56], using 10,000 permutations in ARLEQUIN. Genotypes for each individual at the GnRH and S7 introns were compiled and used to calculate *F*_ST _for the multi-locus dataset and for pairwise comparisons between locations in ARLEQUIN. The false discovery rate among multiple comparisons was controlled as described above. Median-joining networks for alleles at each locus were constructed using the program NETWORK. We tested for correlation between pairwise *F*_ST _values and geographic distance (isolation-by-distance) among all populations using the Isolation-by-Distance Web Service [60] as described above.

#### Migration

Migration rates between groups (*Nm*: where *N *is effective population size and *m *is migration rate) were calculated with the software MIGRATE 3.1.6 [64,65]. To minimize the parameters run, we pooled locations that showed no pairwise structure (i.e. those locations with a pairwise Φ_ST _that did not significantly differ from zero) into demes defined by region (see Results). This program uses a Bayesian MCMC search strategy of a single, replicated, two million step chain. The default settings for priors were used with an unrestricted migration model. Estimates of the number of immigrants per generation (*Nm*) were calculated by multiplying final estimates of θ and M [66].

## Results

### Mitochondrial DNA

**Figure 2:**
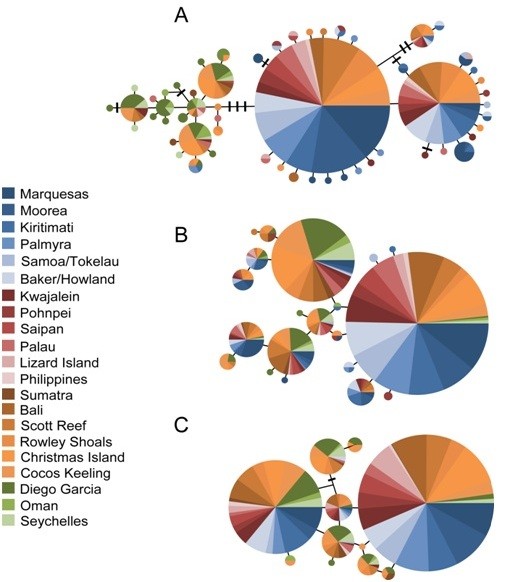
**Median-joining networks for *Cephalopholis argus***. Networks were constructed using the program NETWORK 4.5 [43] for (a) 550 cytochrome *b *sequences (b) alleles at GnRH intron from 488 individuals, and (c) alleles at S7 intron for 490 individuals. Each circle represents one mitochondrial haplotype or nuclear allele with the area of each circle proportional to the number of that particular haplotype or allele in the dataset; dashes represent hypothetical haplotypes or alleles; colors represent collection location (see key).

**Figure 3:**
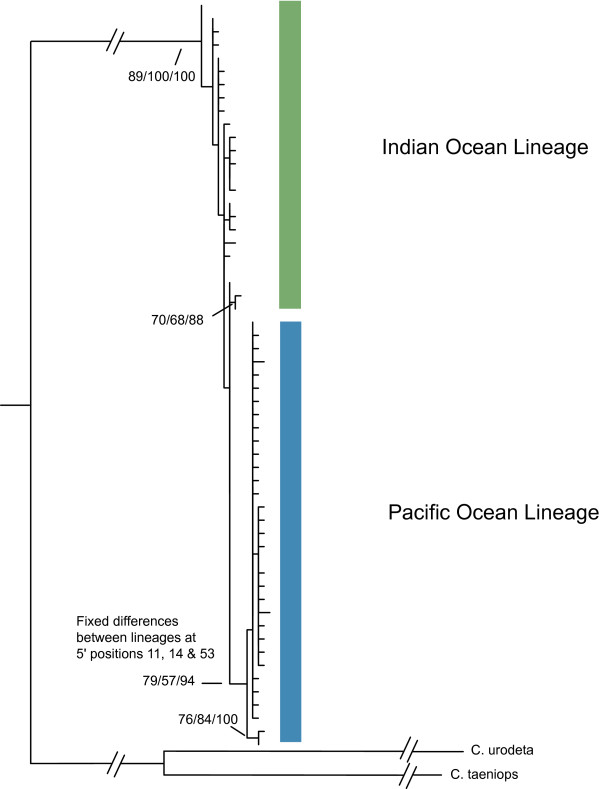
**Phylogenetic tree of C*ephalopholis argus *cytochrome *b *haplotypes**. The best maximum likelihood tree generated using program default settings in RAxML [44] and rooted using two congenerics (*C. urodeta *and *C. taeniops*). Bootstrap support values were calculated using default settings with 1000 replicates. For comparison neighbor-joining bootstrap values (1000 bootstrap replicates) and Bayesian posterior probabilities are presented. Colored bars delineate the Pacific and Indian Ocean lineages separated by three fixed differences (see figure 1).

We resolved a 729 bp segment of cytochrome *b *in 550 individuals yielding 57 haplotypes with 34 of these haplotypes observed in single individuals (Table 1). Due to geographic proximity and a lack of genetic differentiation (as measured by pairwise Φ_ST_) we grouped the specimens from the central Pacific locations of Samoa and Tokelau, and Baker and Howland Island. The number of individuals (N), number of haplotypes (N_h_), haplotype diversity (*h*), and nucleotide diversity (π) for each location are provided in Table 1. Overall nucleotide diversity in *C. argus *was low (π = 0.005) while the corresponding haplotype diversity was high (*h *= 0.80). Across all samples, π = 0.001 - 0.009 and *h *= 0.38 - 0.96 with higher genetic diversity detected in the Pacific compared to the Indian Ocean (unpaired *t*-test, π: t = 2.22, df = 19, *P *= 0.039; *h*: t = 2.88, df = 19, *P *= 0.010). Using the program BEAST and implementing a molecular clock of 2% per million years [67-69] we estimated a coalescence time of approximately 930,000 years with bounds of the 95% highest posterior density intervals (HPD) of 0.5 and 1.5 million yrs, dates that correspond to the middle of the Pleistocene. The median-joining network revealed two clusters of haplotypes that are distinguished by three substitutions (*d *= 0.008, Figure 2a). The two lineages, which we refer to as the Pacific and Indian Ocean lineages, were confirmed by the phylogenetic analyses (Figure 3). Coalescence times for the two lineages were 580,000 (95% HPD = 0.25 - 1.0 million) and 520,000 (95% HPD = 0.22 - 0.88 million) yrs, respectively. No haplotypes from the Pacific Ocean lineage were detected at the western Indian Ocean locations of Diego Garcia, Oman, and Seychelles while 10 of 291 samples from the Pacific Ocean fell into the Indian Ocean lineage (Figure 1). A region of extensive overlap was found at the Indian Ocean locations of Bali, Scott Reef, Rowley Shoals, Christmas Island, and Cocos/Keeling Islands (Figure 1).

Overall Φ_ST _was 0.297 (*P *< 0.001). When we grouped samples by ocean basin (as described in Methods) we found significant structure between the Pacific and Indian Oceans (Φ_CT _= 0.242, *P *< 0.001). Within oceans we found low but significant structure in the Pacific Ocean (Φ_ST _= 0.036, *P *< 0.001) and higher structure in the Indian Ocean (Φ_ST _= 0.249, *P *< 0.001). Pairwise comparisons indicate low levels of structure at the eastern edge of the range distinguishing Marquesas and Moorea (Table 2) but no structure across the entire central Pacific from Kiritimati to Lizard Island (Table 2). While there was no structure among locations in the western Indian Ocean (Diego Garcia, Oman, and Seychelles) and Sumatra, high levels of structure were observed between these locations and the eastern Indian Ocean (Christmas Island, Cocos/Keeling, Bali, Scott Reef, and Rowley Shoals).

**Table 2:** Pairwise *F *statistics for 21 populations of *Cephalopholis argus*

Location	1	2	3	4	5	6	7	8	9	10	11	12	13	14	15	16	17	18	19	20	21
1. Marquesas	-	0.010	0.020	0.031	**0.077**	0.048	0.015	0.038	0.024	-0.001	-0.077	-0.018	**0.093**	0.021	**0.059**	**0.089**	**0.197**	**0.298**	**0.335**	**0.193**	**0.255**
2. Moorea	0.042	-	-0.021	0.004	0.035	0.023	0.003	0.026	0.004	0.007	-0.117	0.117	**0.095**	0.022	**0.064**	**0.093**	**0.200**	**0.399**	**0.340**	**0.188**	**0.261**
3. Kiritimati	0.039	0.059	-	0.002	0.017	-0.020	-0.029	0.036	-0.007	0.004	-0.044	0.041	**0.222**	0.026	**0.111**	**0.122**	**0.269**	**0.380**	**0.424**	**0.312**	**0.374**
4. Palmyra	0.036	0.021	0.006	-	-0.002	-0.027	-0.019	**0.088**	0.017	0.024	-0.077	0.161	**0.275**	**0.067**	**0.164**	**0.164**	**0.322**	**0.496**	**0.471**	**0.381**	**0.431**
5. Samoa/Tokelau	0.022	0.044	-0.002	0.002	-	0.004	0.012	**0.108**	0.039	0.057	-0.115	0.162	**0.277**	**0.098**	**0.190**	**0.183**	**0.329**	**0.483**	**0.476**	**0.372**	**0.426**
6. Baker/Howland	0.063	**0.104**	-0.023	0.032	-0.006	-	-0.004	**0.089**	0.027	0.041	-0.125	0.025	**0.241**	**0.077**	**0.164**	**0.169**	**0.309**	**0.361**	**0.457**	**0.341**	**0.399**
7. Kwajalein	**0.114**	**0.135**	0.015	0.030	0.059	0.036	-	0.032	-0.001	-0.001	-0.133	0.022	**0.151**	0.026	**0.097**	**0.104**	**0.229**	**0.328**	**0.381**	**0.242**	**0.304**
8. Pohnpei	0.019	0.015	-0.025	-0.014	-0.034	-0.019	0.029	-	0.050	0.002	-0.063	0.073	0.009	0.004	0.021	0.007	**0.079**	**0.280**	**0.225**	0.067	**0.135**
9. Saipan	**0.109**	**0.107**	-0.011	0.040	0.055	0.015	0.009	0.054	-	0.015	-0.089	0.048	**0.169**	0.017	**0.077**	**0.118**	**0.233**	**0.355**	**0.372**	**0.244**	**0.300**
10. Palau	**0.097**	**0.103**	0.013	0.002	0.056	0.050	-0.020	0.050	0.006	-	0.033	0.035	0.060	0.004	0.041	0.045	**0.145**	**0.286**	**0.292**	0.138	**0.202**
11. Lizard Is	0.008	-0.011	0.009	-0.015	-0.009	0.036	0.052	-0.020	0.040	-0.111	-	0.128	0.174	-0.045	0.040	0.033	**0.187**	**0.432**	**0.348**	0.222	**0.290**
12. Philippines	0.140	0.205	-0.039	-0.003	0.087	0.020	-0.095	0.072	-0.088	-0.093	0.070	-	0.043	0.003	-0.010	0.047	0.046	0.160	**0.147**	0.107	0.082
13. Bali	**0.169**	**0.179**	0.072	0.063	**0.126**	**0.112**	-0.015	0.096	0.039	-0.014	0.092	-0.077	-	**0.055**	0.006	-0.024	0.001	**0.151**	**0.093**	-0.028	0.022
14. Scott Reef	**0.103**	**0.085**	0.007	0.030	0.057	0.035	-0.001	0.050	-0.027	-0.012	0.042	-0.095	0.017	-	0.012	**0.046**	**0.124**	**0.224**	**0.257**	**0.115**	**0.177**
15. Rowley Shoals	**0.165**	**0.156**	0.062	0.067	**0.119**	**0.099**	0.003	0.100	0.022	-0.010	0.094	-0.084	-0.016	0.017	-	0.024	**0.059**	**0.137**	**0.155**	0.035	**0.084**
16. Christmas Is	**0.271**	**0.263**	**0.169**	**0.167**	**0.224**	**0.206**	0.067	**0.196**	**0.116**	0.064	**0.190**	-0.004	0.014	**0.095**	0.014	-	0.020	**0.168**	**0.134**	0.010	0.057
17. Cocos/Keeling	**0.391**	**0.387**	**0.287**	**0.276**	**0.345**	**0.327**	**0.150**	**0.303**	**0.226**	**0.158**	**0.294**	0.105	0.081	**0.201**	**0.092**	0.009	-	0.030	**0.041**	-0.027	-0.007
18. Sumatra	**0.830**	**0.906**	**0.731**	**0.751**	**0.849**	**0.805**	**0.535**	**0.835**	**0.676**	**0.595**	**0.836**	0.570	0.461	**0.615**	**0.459**	0.277	0.147	-	-0.019	-0.045	-0.008
19. Diego Garcia	**0.765**	**0.798**	**0.694**	**0.703**	**0.759**	**0.737**	**0.565**	**0.738**	**0.659**	**0.604**	**0.734**	**0.644**	**0.511**	**0.611**	**0.498**	**0.351**	**0.228**	0.039	-	0.014	-0.009
20. Oman	**0.790**	**0.861**	**0.695**	**0.718**	**0.801**	**0.760**	**0.521**	**0.620**	**0.648**	**0.580**	**0.775**	**0.379**	**0.472**	**0.592**	**0.453**	**0.299**	0.174	-0.034	-0.013	-	-0.028
21. Seychelles	**0.778**	**0.831**	**0.688**	**0.706**	**0.775**	**0.742**	**0.521**	**0.741**	**0.640**	**0.582**	**0.738**	**0.595**	**0.475**	**0.596**	**0.476**	**0.337**	**0.216**	0.109	-0.005	0.055	-

Pairwise Φ_ST _values for cytochrome *b *data are below diagonal and pairwise *F*_ST _values for multi-locus nuclear dataset are above diagonal. Significant comparisons are in bold. We maintained α = 0.05 among all pairwise tests by controlling for the false discovery [51,52]. The corrected α = 0.008.

**Figure 4:**
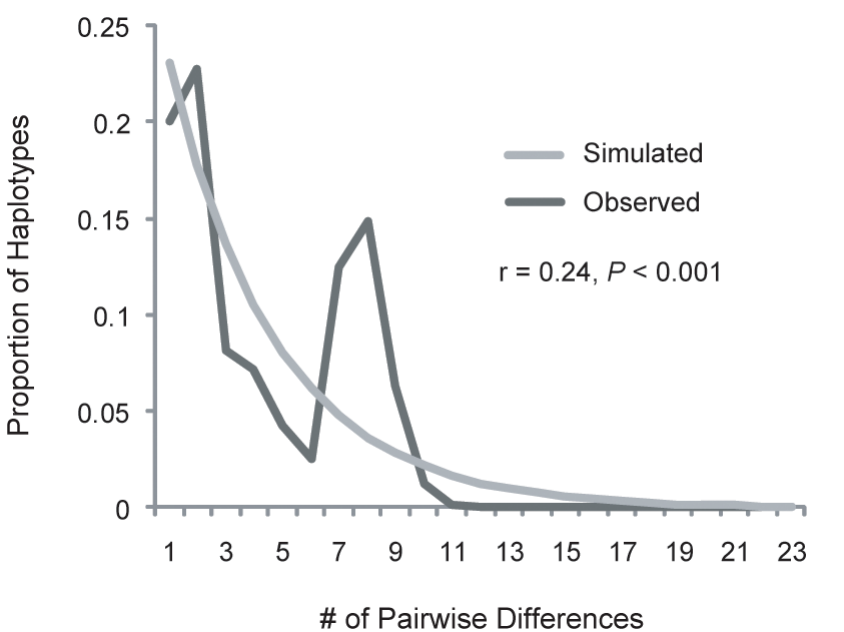
**Mismatch distribution for *Cephalopholis argus***. Mismatch distribution based on 550 cytochrome *b *sequences from twenty-one populations. The dark colored line represents the observed and light colored line is the simulated pairwise differences as reported by DnaSP 5.0 [39]. The Harpending's raggedness index as calculated in ARLEQUIN 3.5 [42] and corresponding *P*-value are shown.

Population expansion parameters for the overall dataset gave conflicting results. As expected with the presence of two divergent mitochondrial lineages, the mismatch distribution for the overall dataset was bimodal (Figure 4) and resulted in a significant raggedness index (r = 0.24, *P *< 0.001), a result that indicates a stable population. In contrast, Fu's *F*_S _resulted in *F*_S _= -12.8 (*P *< 0.001) signifying an excess of low-frequency haplotypes and an expanding population. Grouping locations that demonstrated no significant population structure (see Table 2) resulted in five groups: French Polynesia (FP) = Marquesas and Moorea; central-west Pacific (CW) = Kiritimati, Palmyra, Samoa/Tokelau, Baker/Howland, Kwajalein, Pohnpei, Saipan, Palau, Lizard Island, Philippines, and Scott Reef; Indo-Pacific boundary (IB) = Bali and Rowley Shoals; eastern Indian Ocean (EI) = Cocos/Keeling and Christmas Islands; western Indian Ocean (WI) = Sumatra, Diego Garcia, Oman, and Seychelles. Despite their close geographic proximity and lack of genetic structure with many populations in the CW, Bali and Rowley Shoals were grouped separately because they demonstrate significant genetic structure when compared to Samoa/Tokelau and Baker/Howland. When analyzed separately, mismatch analyses resulted in non-significant raggedness indices for each group (data not presented). Fu's *F*_S _indicated expanding populations for FP (*F*_S _= -4.8, *P *= 0.014), CW (*F*_S _= -20.9, *P *< 0.001) and WI (*F*_S _= -8.9, *P *< 0.001) but no evidence for population expansion was found for either IB (*F*_S _= -0.43, *P *= 0.48) or EI (*F*_S _= -0.48, *P *= 0.63).

### Nuclear introns

We resolved 245 bp of the GnRH intron in 488 specimens and 393 bp of the S7 intron in 490 specimens (Table 1). Seven polymorphic sites yielded 11 alleles at the GnRH locus and 15 polymorphic sites yielded 20 alleles at the S7 locus. Median-joining networks for the GnRH and S7 introns revealed two prominent alleles at each locus that were found throughout the species' range (Figure 2b, c). The number of individuals (*N*), number of alleles (*N*_a_), observed heterozygosity (*H*_O_), expected heterozygosity (*H*_E_), and the corresponding *P*-value for the exact tests for HWE are listed in Table 1. The samples from the Marquesas and Samoa/Tokelau were found to be inconsistent with HWE expectations with an excess of homozygotes at the GnRH locus (*P *= 0.002 and 0.044, respectively) while the sample from Diego Garcia was found to have an excess of heterozygotes at the S7 locus (*P *= 0.047) (Table 1). Across all samples *H*_E _= 0.26 - 0.80 for the GnRH intron and *H*_E _= 0.28 - 0.79 for the S7 intron with higher values of *H*_E _detected in the Pacific compared to the Indian Ocean (unpaired *t*-test, GnRH: t = 3.17, df = 19, *P *= 0.005; S7: t = 3.99, df = 19, *P *< 0.001). There was no indication of linkage disequilibrium between the two loci (*P *> 0.05).

Overall *F*_ST _values for GnRH, S7, and the multi-locus dataset were *F*_ST _= 0.078 (*P *< 0.001), *F*_ST _= 0.099 (*P *< 0.001), and *F*_ST _= 0.127 (*P *< 0.001), respectively. Analyses of these data reveal patterns of population structure that are concordant with the mitochondrial dataset. Grouping samples by ocean basin (as above) revealed significant structure between the Pacific and Indian Oceans (GnRH, *F*_CT _= 0.056, *P *= 0.002; S7, *F*_CT _= 0.103, *P *< 0.001, multi-locus *F*_CT _= 0.154, *P *< 0.001) and significant structure within ocean basins (Pacific Ocean: GnRH, *F*_ST _= 0.020, *P *= 0.025; S7, *F*_ST _= 0.041, *P *< 0.001; multi-locus, *F*_ST _= 0.013, *P *= 0.039; Indian Ocean: GnRH, *F*_ST _= 0.074, *P *< 0.001; S7, *F*_ST _= 0.049, *P *< 0.001; multi-locus, *F*_ST _= 0.072, *P *< 0.001). Pairwise *F*_ST _for the multi-locus dataset are reported in Table 2. Overall the nuclear dataset measured lower levels of population structure compared to mtDNA. Using the multi-locus dataset we found little population subdivision across the central Pacific and no structure in the western Indian Ocean. This dataset did not detect the low levels of population structure at the Marquesas and Moorea as revealed in the mtDNA dataset, nor were the Indian Ocean populations as divergent using these markers (Table 2).

As might be expected from loci with low numbers of closely related alleles, the mismatch distributions for the overall nuclear dataset and for the five geographic groups (FP, CW, IB, EI, and WI) were unimodal and resulted in non-significant raggedness indices (overall dataset: GnRH, r = 0.32, *P *= 0.082; S7, r = 0.12, *P *= 0.235; data for geographic groups not shown). Fu's *F*_S _calculations offered no evidence for expanding populations for either the overall dataset (GnRH, *F*_S _= -0.34, *P *= 0.521; S7, *F*_S _= -3.93, *P *= 0.162) or for the geographic groups (data not shown).

### Migration

**Figure 5:**
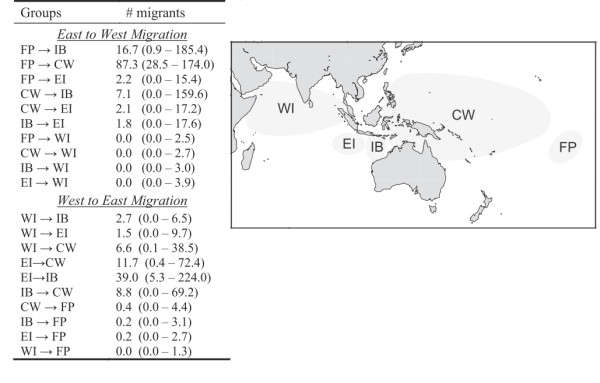
**Migration rates for *Cephalopholis argus***. Migration rates (*Nm*: where *N *is effective female population size and *m *is migration rate) based on cytochrome *b *sequences calculated using MIGRATE 3.1.6 [54,55]. Locations with non-significant pairwise Φ_ST _values were grouped (see Table 2). French Polynesia (FP) = Marquesas and Moorea; central-west Pacific (CW) = Kiritimati, Palmyra, Samoa/Tokelau, Baker/Howland, Kwajalein, Pohnpei, Saipan, Palau, Lizard Island, Philippines, and Scott Reef; Indo-Pacific boundary (IB) = Bali and Rowley Shoals; eastern Indian Ocean (EI) = Christmas Island and Cocos/Keeling; western Indian Ocean (WI) = Sumatra, Diego Garcia, Oman, and Seychelles. The direction of migration is indicated. Numbers of migrants per generation between geographic regions are reported with 95% confidence intervals in parentheses.

Migration analyses for the nuclear dataset proved to be uninformative. Posterior probabilities did not narrow on a single mode for several comparisons and confidence intervals were unreasonably large. We present only the mtDNA data here. Migration rates indicate that while the populations of *C. argus *at the ends of the range (FP and WI) contribute to genetic diversity across the central portion of the range (*Nm *per generation = 2.2 - 87.3 and 1.5 - 6.6, respectively), they rarely receive migrants (*Nm *per generation = 0.0 - 0.4 and 0.0, respectively; Figure 5). There is evidence of considerable migration between the other groups (*Nm *per generation = 1.8 - 39.0; Figure 5).

### Isolation by distance (IBD)

Mantel tests showed a strong correlation between genetic distance (Φ_ST _or *F*_ST_) and geographic distance in the mtDNA (r = 0.749, *P *= 0.001) and the multi-locus nuclear (r = 0.715, *P *< 0.001) datasets. Replacing negative values of Φ_ST _and *F*_ST _with zeros did not affect the pattern or statistical significance. To test if genetic structure between ocean basins was driving IBD we conducted Mantel tests within oceans and found weaker but still significant correlations between genetic distance and geographic distance with the Cyt*b *dataset (Pacific Ocean: r = 0.301, *P *= 0.033; Indian Ocean: r = 0.778, *P *= 0.004) but not the multi-locus nuclear dataset (Pacific Ocean: r = -0.056, *P *= 0.629; Indian Ocean: r = 0.315, *P *= 0.085).

## Discussion

The origin of the remarkable species richness of the Coral Triangle (CT) has fostered numerous and seemingly conflicting hypotheses. The center of origin hypothesis postulates that elevated rates of speciation in the CT have resulted in high species diversity [9]. In contrast, the center of accumulation hypothesis contends that taxa have evolved peripherally and subsequently accumulate in the CT due to prevailing currents [19]. Finally, the region of overlap hypothesis states that the observed pattern is the result of admixture of the distinct biotas of the Pacific and Indian Oceans [21]. Despite considerable effort to determine the mechanism driving species diversity in the Indo-Pacific, no consensus has emerged [7,24,25]. Our genetic survey of *C. argus *across 18,000 km of the Indo-Pacific lends some insight into this debate.

*Cephalopholis argus *demonstrates significant levels of genetic structure that indicate a historical partition between the Pacific and Indian Oceans (Table 2). Two mitochondrial lineages are distinguished by fixed differences (*d *= 0.008) indicating isolation for approximately one million years (95% HPD intervals are 0.5 - 1.5 million yrs), a time interval that corresponds to Pleistocene sea level fluctuations linked to Milankovitch climate cycles on the scale of 10^5 ^years [70]. Our analyses indicate expanding populations with migration toward the center of the range. The high genetic diversity of this species within and adjacent to the CT is a result of mixing Pacific and Indian Ocean lineages (Figures 1, 5). Hence these data support isolation of Pacific and Indian Ocean populations during prolonged and repeated sea level fluctuations of the Pleistocene, followed by population expansion and colonization of the CT from both the Pacific and Indian Oceans: a pattern that is consistent with predictions of the region of overlap hypothesis.

While incomplete lineage sorting is a serious problem for species level reconstructions, our pattern of divergence across the IPB is corroborated by three independent markers. Additionally, the finding of isolation by distance across the species range is strong evidence that the patterns we present here are not driven by stochastic events.

### Indo-Pacific Barrier-the mechanism of isolation

**Figure 6:**
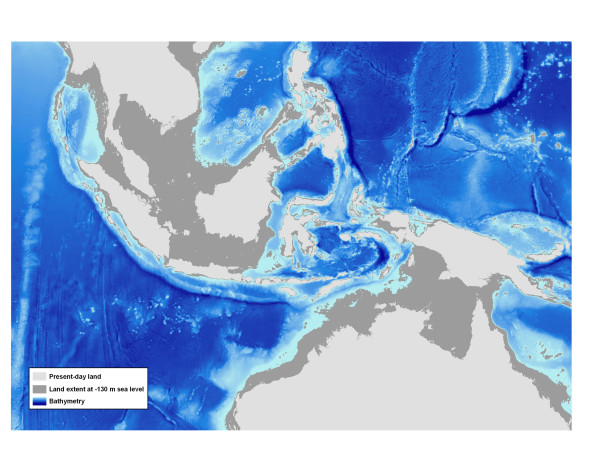
**Map of Indo-Malaysia region during glacial maxima**. Map shows the effect of lowered sea level on habitat in the region during Pleisotocene glacial maxima (Figure credit: Eric Franklin).

The Sunda shelf, surrounding the Malay Peninsula and western islands of Indonesia, and the Sahul shelf off northern Australia and New Guinea, separate the Pacific and Indian Oceans and together are known as the Indo-Pacific Barrier (IPB) [71]. Over the last 700,000 yrs there have been at least three to six glacial cycles that lowered sea level as much as 130 m below present levels (Figure 6, [23,72-74]). Species on the continental shelves were repeatedly subjected to widespread extirpations and presumably interruption of gene flow between Pacific and Indian Ocean populations. However, at glacial maxima the isolation of the two ocean basins was not complete. Associated with the change in sea level were concomitant changes in oceanographic current patterns, altered discharge of local rivers, with corresponding changes in temperature and salinity [75,76]. The narrow seaways that remained were likely under the influence of cooler upwelling, further limiting the availability of suitable habitat for tropical marine organisms [10,23,71,73,77]. In sum, the isolating mechanism between ocean basins may have been due to both ecological and geological factors.

The evidence for the impact of the IPB on shallow tropical marine organisms is extensive and compelling. Historical and contemporary restrictions to dispersal between the Pacific and Indian Oceans are indicated by the confinement of many demersal species primarily to one ocean or the other [3,21,78,79]. More recently, genetic data have been used to assess the IPB. Studies of demersal organisms that lack vagile adults have found intraspecific genetic differentiation across the IPB in many fishes [80-88] and invertebrates [89-96] with few exceptions [67,69,95,97,98]. Genetic analyses reveal signatures of isolation that correspond to Pleistocene sea level fluctuations across a diversity of taxa [82,85,93,97,99] including *C. argus*. This species demonstrates strong population structure between Pacific and Indian Ocean locations in both the mitochondrial and nuclear datasets. The mismatch distribution for *C. argus *is distinctly bimodal (Figure 4) which is characteristic of species under the influence of a strong biogeographic barrier [100,101]. The mid-Pleistocene age of the two mitochondrial lineages of *C. argus *coupled with assortment by ocean basins is compelling evidence that the divergence is a result of isolation on either side of the IPB.

### Eastern Indian Ocean and the Coral Triangle: A region of overlap

Since the last glacial maximum about 18,000 yrs ago, the land bridge that impeded dispersal between the Pacific and Indian oceans submerged and the rising sea level not only opened dispersal pathways but was also accompanied by an approximately 10 fold increase in suitable shallow reef habitat [4]. Woodland [21] was the first to propose that range expansions of species formed in isolation during Pleistocene glacial cycles contributed to the incredible species richness of the CT. His work on species distributions of rabbitfishes (Family Siganidae) and later the work of Donaldson [102] on hawkfishes (Family Cirrhitidae) offer supporting evidence. Range expansions are also indicated by the presence of a hybrid zone in the eastern Indian Ocean [103]. Cocos/Keeling and Christmas Islands lie 500 and 1,400 km, respectively, from the southern coast of the Indonesian Island of Java, and are a known region of overlap for Pacific and Indian Ocean fish faunas. Here, sister species that are otherwise restricted to different oceans inhabit the same reefs and in many cases hybridize [103-105]. Notably, we found nearly equal proportions of Pacific and Indian Ocean *C. argus *haplotypes in this hybrid zone (Figure 1). These findings demonstrate that, at least in terms of intraspecific genetic diversity, the introgression is not restricted to Cocos/Keeling and Christmas Islands but instead extends well into Indonesia, the western Pacific, and to a lesser extent, the central Pacific.

If we provisionally assume that genetic divergences are the result of isolation across the IPB, we can estimate the degree of introgression since the last ice age. In some taxa, effective migration between ocean basins is absent as evidenced by a lack of shared haplotypes between oceans (*Chlorurus sordidus *[82]; *Penaeus monodon *[93]). Other taxa reveal signatures of historical isolation but lack contemporary spatial structure (*Naso brevirostris *[97]). Pacific and Indian Ocean populations of *C. argus *share haplotypes but mixing is incomplete as evidenced by significant population structure between oceans, a pattern observed in several other species (*Myripristis berndti *[84]; *Naso vlamingii *[99]; *Nerita albieilla *[106]). *C. argus *is unique in that it demonstrates unidirectional dispersal out of the western Indian Ocean (WI) and French Polynesia (FP) toward the center of the range (Figure 5) while populations in the CT and western Australia, the area near the Indo-Pacific boundary, demonstrate high levels of bidirectional dispersal, high genetic diversity, and extensive lineage overlap (Figures 1, 5).

There is compelling evidence for the influence of the IPB on coral reef organisms from intraspecific lineage sorting to species level distributions. The degree of range expansion or lineage mixing after the last glacial maximum varies among taxa and may reflect species level differences in dispersal ability, reproductive strategy, competitive ability, or habitat requirements.

### Phylogeographic inferences: emerging patterns in Indo-Pacific reef fishes

Our dataset allows for several phylogeographic inferences. Molecular diversity indices and the topology of the medium joining networks indicate that Indian Ocean populations harbor more genetic diversity. The position of the Indian Ocean lineage in the phylogenetic tree indicates that this lineage may be older but coalescence dates do not support this. Taken together these data may indicate that during low sea level stands, populations in the western Indian Ocean were less severely impacted than those in the Pacific. *C. argus *demonstrates no population structure across the nearly 8,000 km central Pacific range, from Kiritimati to Palau. However, pairwise Φ_ST _and *F*_ST _values and migration rates indicate that populations at the eastern end of the range at Moorea and the Marquesas are isolated. This pattern of extensive population connectivity across the central Pacific with isolation at the ends of the Pacific range is emerging in reef fishes (reviewed in [86]; see also [107]).

### Biogeographic inferences: the Western Indian Ocean Province

The isolation of the western Indian Ocean (WIO) is supported by both species distributions [22] and intraspecific genetic data [84,87,97], evidence that the microevolutionary divergences documented with DNA sequence data can lead to macroevolutionary partitions between species. Genetic analyses seperate Indian Ocean populations of *C. argus *along an east-west gradient and indicate unidirectional dispersal out of the WIO. Despite being geographically located in the Indian Ocean, the eastern Indian Ocean faunas at Cocos/Keeling and Christmas Islands, and Western Australia are closely affiliated with the Pacific ichthyofauna with only 5% of reef fishes at Cocos/Keeling of exclusively Indian Ocean origin [108]. Instead, these islands are considered to be a part of the Indo-Polynesian Province that stretches from the eastern Indian Ocean to French Polynesia [3,22]. Diego Garcia in the Chagos Archipelago lies in the middle of the Indian Ocean 1,900 km east of the Seychelles and 2,400 km west of Cocos/Keeling. Fish surveys in the Chagos Islands delineated the archipelago into two distinct assemblages, with the northern portion sharing affinities with the eastern Indian Ocean and the southern portion (including Diego Garcia) more closely aligned with faunal assemblages further west [109]. The distinction of the ichthyofauna assemblage of the southern Chagos Archipelago coupled with a lack of intraspecific genetic structure in two species of reef fishes from Diego Garcia and sites further west (*Lutjanus kasmira *[98] and *C. argus*, this study) indicate that Diego Garcia is a part of the Western Indian Ocean Province as described by Briggs [22]. Faunal surveys indicate that the fishes of India and Sri Lanka have a strong affiliation with the Malay Peninsula [22]. Taken together, these data indicate that the western boundary of the Indo-Polynesian Province lies east of Oman and includes India, Sri Lanka, and the northern Chagos Archipelago [110]. While we use species distributions and genetic data to define biogeographic provinces, the mechanisms that separate the eastern and western Indian Ocean are unknown and require more detailed genetic and oceanographic work.

## Conclusions

Our genetic survey of the grouper *Cephalopholis argus *indicates that this species was strongly impacted by Pleistocene sea level fluctuations which resulted in the partitioning of this species into Pacific and Indian Ocean mitochondrial lineages that are distinguished by fixed differences (*d *= 0.008). Following the end of the last glacial maximum, connectivity between the Pacific and Indian Oceans resumed and *C. argus *populations expanded. Representatives of each mitochondrial lineage are now found in both oceans with the center of diversity occurring in the Coral Triangle: a pattern that we offer as support for the region of overlap hypothesis. In a recent review 15 out of 18 species demonstrated significant structure across the IPB, such that subsequent contact would constitute support of the region of overlap hypothesis [98]. However, the studies cited above, on a diverse array of marine taxa, offer equally compelling evidence for the other two competing hypotheses: the center of origin and center of accumulation. None of these hypotheses are mutually exclusive, and acting in concert, they are likely to explain the patterns of biodiversity in the Indo-Pacific.

## Authors' contributions

MRG obtained samples from the central Pacific and the Philippines, participated in DNA sequencing, data analysis, data interpretation, and prepared the manuscript. BWB participated in study design, funding, and sampling from the Pacific and Indian Oceans, and helped prepare the manuscript. TRB participated in DNA sequencing. LAR obtained samples from across the Pacific and Indian Oceans, and helped prepare the manuscript. SJN obtained samples from Western Australia and the eastern Indian Ocean. LAG and LVH participated in sequencing individuals from Western Australia and the east Indian Ocean. MTC obtained samples from throughout the Pacific and Indian Oceans, participated in the design and coordination of the study, and helped prepare the manuscript. All authors approved the final manuscript.

## References

[B1] Bowen BW Roman J Gaia's handmaidens: the Orlog model for conservation biology Con Biol 2005 19 1037 1043 10.1111/j.1523-1739.2005.00100.x

[B2] Veron JEM Corals in space and time: The biogeography and evolution of the Scleratinia 1995 Sydney: University of New South Wales Press

[B3] Randall JE Zoogeography of shorefishes of the Indo-Pacific region Zool Studies 1998 37 227 268

[B4] Bellwood DR Wainwright PC Sale PF The history and biogeography of fishes on coral reefs Coral reef fishes: dynamics and diversity in a complex ecosystem 2002 San Diego: Academic Press 5 32

[B5] Roberts CM McClean CJ Veron JEN Hawkins JP Allen GR McAllister DE Mittermeier CG Schueler FW Spalding M Wells F Vynne C Werner TB Marine biodiversity hotspots and conservation priorities for tropical reefs Science 2002 295 1280 1284 10.1126/science.1067728 11847338

[B6] Briggs JC Marine centers of origin as evolutionary engines J Biogeogr 2003 30 1 18 10.1046/j.1365-2699.2003.00810.x

[B7] Mora C Sale PF Kritzer JP Ludsin SA Patterns and processes in reef fish diversity Nature 2003 421 933 936 10.1038/nature01393 12606998

[B8] Allen GR Conservation hotspots of biodiversity and endemism for Indo-Pacific coral reef fishes 2007 18 Aquatic Conservation: Marine and Freshwater Ecosystems 541 556

[B9] Ekman S Zoogeography of the sea 1986 London: Sidgwick and Jackson

[B10] McManus JW Gabrie C, Vivien MH Marine speciation, tectonics, and sea-level changes in southeast Asia Proceedings of the Fifth International Coral Reef Congress 1985 Moorea: Antenne Museum-Ephe 133 138 27 May-1 June 1985; Tahiti

[B11] Briggs JC The marine East Indies: diversity and speciation J Biogeogr 2005 32 1517 1522 10.1111/j.1365-2699.2005.01266.x

[B12] Rocha LA Bowen BW Speciation in coral reef fishes J Fish Biol 2008 72 1101 1121 10.1111/j.1095-8649.2007.01770.x

[B13] Barber PH Erdmann MV Palumbi SR Comparative phylogeography of three codistributed stomatopods: origins and timing of regional lineage diversification in the Coral Triangle Evolution 2006 60 1825 1839 17089967

[B14] DeBoer TS Subia MD Erdmann MV Kovitvongsa K Barber PH Phylogeography and limited genetic connectivity in the endangered giant boring clam across the Coral Triangle Conserv Biol 2008 22 1255 1266 10.1111/j.1523-1739.2008.00983.x 18637905

[B15] Knittweis L Kräemer WE Timm J Kochzius M Genetic structure of *Heliofungia actiniformis *(Scleractinia: Fungiidae) populations in the Indo-Malay Archipelago: implications for the live coral trade management efforts Conserv Biol 2009 10 241 249

[B16] Kochzius M Seidel C Hauschild J Kirchhoff S Mester P Meyer-Wachsmuth I Nuryanto A Timm J Genetic population structure of the blue starfish *Linckia laevigata *and its gastropod ectoparasite *Thyca crystallina* Mar Eco Prog Ser 2009 396 211 219

[B17] Nuryanto A Kochzius M Highly restricted gene flow and deep evolutionary lineages in the giant clam *Tridacna maxima* Coral Reefs 2009 28 607 619 10.1007/s00338-009-0483-y

[B18] Timm J Kochzius M Geological history and oceanography of the Indo-Malay Archipelago shape the genetic population structure in the false clown anemonefish (*Amphiprion ocellaris*) Mol Ecol 2008 17 3999 4014 10.1111/j.1365-294X.2008.03881.x 19238702

[B19] Ladd HS Origin of the Pacific Island molluscan fauna Am J Sci 1960 258A 137 150

[B20] Jokiel P Martinelli FJ The vortex model of coral reef biogeography J of Biogeogr 1992 19 449 458 10.2307/2845572

[B21] Woodland DJ Zoogeography of the Siganidae (Pisces): an interpretation of distribution and richness patterns Bull Mar Sci 1983 33 713 717

[B22] Briggs JC Marine zoogeography 1974 New York: McGraw-Hill

[B23] Voris HK Maps of Pleistocene sea levels in Southeast Asia: Shorelines, river systems and time durations J Biogeogr 2000 27 1153 1167 10.1046/j.1365-2699.2000.00489.x

[B24] Connolly SR Bellwood DR Hughes TP Indo-Pacific biodiversity of coral reefs: deviations from a mid-domain model Ecology 2003 84 2178 2190 10.1890/02-0254

[B25] Halas D Winterbottom R A phylogenetic test of multiple proposals for the origins of the East Indies coral reef biota J Biogeogr 2009 36 1847 1860 10.1111/j.1365-2699.2009.02103.x

[B26] Wallace CC Lessios HA, Macintyre IG The Indo-Pacific center of coral diversity re-examined at species level Proceedings of the 8th international Coral Reef Symposium 1997 Balboa: Smithsonian Tropical Research Institute 365 370 24-29 June 1997; Panama

[B27] Allen GR Adrim M Coral reef fishes of Indonesia Zool Studies 2003 42 1 72

[B28] Barber PH Bellwood DR Biodiversity hotspots: evolutionary origins of biodiversity in wrasses (Halichoeres: Labridae) in the Indo-Pacific and new world tropics Phylogenet Evol 2005 35 235 253 10.1016/j.ympev.2004.10.004 15737594

[B29] Drew J Barber PH Sequential cladogenesis of the reef fish *Pomacentrus moluccensis *(Pomacentridae) supports the peripheral origin of marine biodiversity in the Indo-Australian archipelago Mol Phylogenet Evol 2009 53 335 339 10.1016/j.ympev.2009.04.014 19401237

[B30] Bowen BW Clark AM Abreu-Grobois FA Chavez A Reichart H Ferl RJ Global phylogeography of the ridley sea turtles (*Lepidochelys *spp.) inferred from mitochondrial DNA sequences Genetica 1998 101 179 189 10.1023/a:1018382415005 9692227

[B31] Eble JA Toonen RJ Sorenson L Basch LV Papastamatiou YP Bowen BW Escaping paradise: larval export from Hawaii in an Indo-Pacific reef fish, the Yellow Tang (*Zebrasoma flavescens*) Mar Ecol Prog Ser 2011 428 245 258 10.3354/meps09083 PMC4260458 25505806

[B32] Palumbi SR Molecular biogeography of the Pacific Coral Reefs 1997 16 S47 S52 10.1007/s003380050241

[B33] Bernardi G Bucciarelli G Costagliola D Robertson DR Heiser JB Evolution of coral reef fish *Thalassoma *spp. (Labridae): 1. Molecular phylogeny and biogeography Mar Biol 2004 144 369 375 10.1007/s00227-003-1199-0

[B34] Avise JC Molecular markers, natural history, and evolution 2004 Sunderland: Sinauer Associates, Inc.

[B35] Randall JE Reef and Shore Fishes of the South Pacific 2005 Honolulu: Sea Grant College Program University of Hawaii

[B36] Shpigel M Fishelson L Territoriality and associated behvaiour in three species of the genus *Cephalopholis *(Pisces: Serranidae) in the Gulf of Aquba, Red Sea J Fish Biol 1999 38 887 896

[B37] Leis JM Sale PF The pelagic phase of coral reef fishes: larval biology of coral reef fishes The Ecology of Fishes on Coral Reefs 1991 San Diego: Academic Press 183 230

[B38] Linderman KC Lee TM Wilson WD Claro R Ault JS Transport of larvae originating in southwest Cuba and the Dry Tortugas: evidence for partial retention in grunts and snappers Proc Gulf Carib Fish Inst 2000 52 253 278

[B39] Seutin G White BN Boag PT Preservation of avian and blood tissue samples for DNA analyses Can J Zool 1991 69 82 92 10.1139/z91-013

[B40] Meeker ND Hutchinson SA Ho L Trede NS Method for isolation of PCR-ready genomic DNA from zebrafish tissues BioTechniques 2007 43 610 614 10.2144/000112619 18072590

[B41] Truett GE Mynatt RL Truett AA Walker JA Warman ML Preparation of PCR-quality mouse genomic DNA with hot sodium hydroxide and Tris (HotSHOT) BioTechniques 2000 29 52 54 10907076 10.2144/00291bm09

[B42] Hassan M Lemaire C Fauvelot C Bonhomme F Seventeen new exon-primed intron-crossing polymerase chain reaction amplifiable introns in fish Mol Ecol Notes 2002 2 334 340 10.1046/j.1471-8286.2002.00236.x

[B43] Chow S Hazama K Universal PCR primers for S7 ribosomal protein gene introns in fish Mol Ecol 1998 7 1247 1263 9734083

[B44] Stephens M Smith NJ Donnelly P A new statistical method for haplotype reconstruction from population data Am J Hum Genet 2001 68 978 989 10.1086/319501 11254454 PMC1275651

[B45] Stephens M Donnelly P A comparison of Bayesian methods for haplotype reconstruction from population genotype data Am J Hum Genet 2003 73 1162 1169 10.1086/379378 14574645 PMC1180495

[B46] Librado P Rozas J DnaSP v5 As software for comprehensive analysis of DNA polymorphism data Bioinformatics 2009 25 1451 1452 10.1093/bioinformatics/btp187 19346325

[B47] Maddison DR Maddison WP MacClade 4: analysis of phylogeny and character evolution 2002 Sunderland: Sinauer Associates 10.1159/000156416 2606395

[B48] Nei M Molecular evolutionary genetics 1987 New York: Columbia University Press

[B49] Excoffier L Lischer HEL Arlequin suite ver 3.5: a new series of programs to perform population genetics analyses under Linux and Windows Mol Ecol Res 2010 10 564 567 10.1111/j.1755-0998.2010.02847.x 21565059

[B50] Bandelt HJ Forster P Röhl A Median-joining networks for inferring intraspecific phylogenies Mol Biol Evol 1999 16 37 48 10331250 10.1093/oxfordjournals.molbev.a026036

[B51] Stamatakis A RAxML-VI-HPC: Maximum likelihood-based phylogenetic analyses with thousands of taxa and mixed models Bioinformatics 2006 22 2688 2690 10.1093/bioinformatics/btl446 16928733

[B52] Tamura K Dudley J Nei M Kamar S MEGA4: molecular evolutionary genetics analysis (MEGA) software version 4.0 Mol Biol Evol 2007 24 1596 1599 10.1093/molbev/msm092 17488738

[B53] Huelsenbeck JP Ronquist F MRBAYES: Bayesian inference of phylogeny Bioinformatics 2001 17 754 755 10.1093/bioinformatics/17.8.754 11524383

[B54] Tamura K Nei M Estimation of the number of nucleotide substitutions in the control region of mitochondrial DNA in humans and chimpanzees Mol Biol Evol 1993 10 512 526 8336541 10.1093/oxfordjournals.molbev.a040023

[B55] Harpending HC Signature of ancient population growth in a low-resolution mitochondrial DNA mismatch distribution Human Biol 1994 66 591 600 8088750

[B56] Fu YX Statistical tests of neutrality of mutations against population growth, hitchhiking, and background selection Genetics 1997 147 915 925 9335623 10.1093/genetics/147.2.915 PMC1208208

[B57] Benjamini Y Yekutieli D The control of the false discovery rate in multiple testing under dependency Annals of Statistics 2001 29 1165 1188 10.1214/aos/1013699998

[B58] Narum SR Beyond Bonferroni: Less conservative analyses for conservation genetics Conservation Genetics 2006 7 783 787 10.1007/s10592-005-9056-y

[B59] Jensen JL Bohonak AJ Kelley ST Isolation by distance, web service BMC Genetics 2005 6 13 15760479 10.1186/1471-2156-6-13 PMC1079815

[B60] Drummond AJ Rambaut A BEAST: Bayesian evolutionary analysis by sampling trees BMC Evol Biol 2007 7 214 221 10.1186/1471-2148-7-214 17996036 PMC2247476

[B61] Guindon S Gascuel O A simple, fast, and accurate algorithm to estimate large phylogenies by maximum likelihood Syst Biol 2003 14 685 695 10.1080/10635150390235520 14530136

[B62] Posada D jModelTest: Phylogenetic model averaging Mol Biol Evol 2008 25 1253 1256 10.1093/molbev/msn083 18397919

[B63] Molecular Evolution, Phylogenetics, and Epidemiology Website http://tree.bio.ed.ac.uk/software/tracer/

[B64] Beerli P Felsenstein J Maximum-likelihood estimation of migration rates and effective population numbers in two populations using a coalescent approach Genetics 1999 152 763 773 10353916 10.1093/genetics/152.2.763 PMC1460627

[B65] Beerli P Felsenstein J Maximum likelihood estimation of a migration matrix and effective population sizes in n subpopulations by using a coalescent approach Proc Nat Acad Sci 2001 98 4563 4568 10.1073/pnas.081068098 11287657 PMC31874

[B66] Beerli P Bertorelle G, Bruford MW, Hauffe HC, Rizzoli A, Vernesi C How to use Migrate or why are Markov chain Monte Carlo programs difficult to use? Population Genetics for Animal Conservation 2009 Cambridge: Cambridge University Press 42 79

[B67] Bowen BW Bass AL Rocha LA Grant WS Robertson DR Phylogeography of the trumpetfish (*Aulostomus *spp.): ring species complex on a global scale Evolution 2001 55 1029 1039 10.1554/0014-3820(2001)055[1029:POTTAR]2.0.CO;2 11430639

[B68] Lessios HA The great American schism: divergence of marine organisms after the rise of the Central American Isthmus Ann Rev Evo Evol Sys 2008 39 63 91

[B69] Reece JS Bowen BW Joshi K Goz V Larson AF Phylogeography of two moray eels indicates high dispersal throughout the Indo-Pacific J Hered 2010 101 391 402 10.1093/jhered/esq036 20375076 PMC2884193

[B70] Hays JD Imbrie J Shackleton NJ Variations in the Earth's Orbit: Pacemaker of the Ice Ages Science 1976 194 1121 1132 10.1126/science.194.4270.1121 17790893

[B71] Fleminger A The Pleistocene equatorial barrier between the Indian and Pacific Oceans and a likely cause for Wallace's line UNESCO Technical Papers in Marine Science 1986 49 84 97

[B72] Chappell J Relative and average sea level changes, and endo-, epi-, and exogenic processes on the earth Sea level, ice, and climatic change Int Ass Hydrol Sci Publ 1981 131 411 430

[B73] Potts DC Evolutionary disequilibrium among Indo-Pacific corals Bull Mar Sci 1983 33 619 632

[B74] Naish T Obliquity-paced Pliocene West Atlantic ice sheet oscillations Nature 2009 458 322 328 10.1038/nature07867 19295607

[B75] Tjia HD Fairbridge RW Java Sea The Encyclopedia of Oceanography 1966 New York: Reinhold 424 429

[B76] Van Andel TH Heath GR Moore TC McGeary DFR Late Quaternary history, climate, and oceanography of the Timor Sea, northwestern Australia Am J Sci 1967 265 737 758 10.2475/ajs.265.9.737

[B77] Galloway RW Kemp EM Keast A Late Cenozoic environments in Australia Ecological Biogeography of Australia 1981 Junk: The Hague 51 80

[B78] McMillan WO Palumbi SR Concordant evolutionary patterns among Indo-West Pacific butterflyfishes Proc Roy Soc B 1995 260 229 236 10.1098/rspb.1995.0085 7784441

[B79] Briggs JC Extinction and replacement in the Indo-West Pacific Ocean J Biogeogr 1999 26 777 783 10.1046/j.1365-2699.1999.00322.x

[B80] Lacson JM Clark S Genetic divergence of Maldivian and Micronesian demes of the damselfishes *Stegastes nigricans*, *Chrysiptera biocellata*, *C. glauca *and *C. leucopoma *(Pomacentridae) Mar Biol 1995 121 585 590 10.1007/BF00349293

[B81] Planes S Fauvelot C Isolation by distance and vicariance drive genetic structure of a coral reef fish in the Pacific Ocean Evolution 2002 56 378 399 11926506 10.1111/j.0014-3820.2002.tb01348.x

[B82] Bay LK Choat JH van Herwerden L Robertson DR High genetic diversities and complex genetic structure in an Indo-Pacific tropical reef fish (*Chlorurus sordidus*): evidence of an unstable evolutionary past? Mar Biol 2004 144 757 767 10.1007/s00227-003-1224-3

[B83] Menezes MR Ikeda M Taniguchi N Genetic variation in skipjack tuna *Katsuwonus pelamis *(L.) using PCR-RFLP analysis of the mitochondrial DNA D-loop region J Fish Biol 2006 68 156 161 10.1111/j.0022-1112.2006.00993.x

[B84] Craig MT Eble JA Robertson DR Bowen BW High genetic connectivity across the Indian and Pacific Oceans in the reef fish *Myripristis berndti *(Holocentridae) Mar Ecol Prog Ser 2007 334 245 254

[B85] Leray M Beldade R Holbrook SJ Schmitt RJ Planes S Bernardi G Allopatric divergence and speciation in coral reef fish: the three-spot *Dascyllus, Dascyllus Trimaculatus*, species complex Evolution 2010 64-5 1218 1230 10.1111/j.1558-5646.2009.00917.x 20002167

[B86] Eble JA Rocha LA Craig MT Bowen BW Not all larvae stay close to home: Long-distance dispersal in Indo-Pacific reef fishes, with a focus on the Brown Surgeonfish (*Acanthurus nigrofuscus*) J Mar Bio 2011 10.1155/2011/518516 PMC4260469 25505914

[B87] Winters KL van Herwerden L Choat HJ Robertson DR Phylogeography of the Indo-Pacific parrotfish *Scarus psittacus*: isolation generates distinctive peripheral populations in two oceans Mar Biol 2010 157 1679 1691 10.1007/s00227-010-1442-4

[B88] Fitzpatrick JM Carlon DB Lippe C Robertson DR The west Pacific hotspot as a source or sink for new species? Population genetic insights from the Indo-Pacific parrotfish *Scarus rubroviolaceus* Mol Ecol 2011 20 219 234 10.1111/j.1365-294X.2010.04942.x 21143329

[B89] Lavery S Moritz C Fielder DR Changing patterns of population structure and gene flow at different spatial scales in *Birgus latro *(the coconut crab) Heredity 1995 74 531 541 10.1038/hdy.1995.75

[B90] Lavery S Mortiz C Fielder DR Indo-Pacific population structure and evolutionary history of the coconut crab *Birgus latro* Mol Ecol 1996 5 557 570 10.1111/j.1365-294X.1996.tb00347.x

[B91] Williams ST Benzie JAH Evidence of a biogeographic break between populations of a high dispersal starfish: congruent regions within the Indo-West Pacific defined by color morphs, mtDNA, and allozyme data Evolution 1998 52 87 99 10.2307/2410923 28568135

[B92] Benzie JAH Major genetic differences between crown-of-thorns starfish (*Acanthaster planci*) populations in the Indian and Pacific Oceans Evolution 1999 53 1782 1795 10.2307/2640440 28565442

[B93] Duda TF Palumbi SR Population structure of the black tiger prawn, *Penaeus monodon*, among western Indian Ocean and western Pacific populations Mar Biol 1999 134 705 710 10.1007/s002270050586

[B94] Barber PH Palumbi SR Erdmann MV Moosa MK A marine Wallace's line? Nature 2000 406 692 693 10.1038/35021135 10963585

[B95] Lessios HA Kessing BD Pearse JS Population structure and speciation in tropical seas: global phylogeography of the sea urchin *Diadema* Evolution 2001 55 955 975 10.1554/0014-3820(2001)055[0955:PSASIT]2.0.CO;2 11430656

[B96] Lessios HA Kane J Robertson DR Phylogeography of the pantropical sea urchin *Tripneustes*: Contrasting patterns of population structure between oceans Evolution 2003 57 2026 2036 14575324 10.1111/j.0014-3820.2003.tb00382.x

[B97] Horne JB van Herwerden L Choat HJ Robertson DR High population connectivity across the Indo-Pacific: congruent lack of phylogeographic structure in three reef fish congeners Mol Phylogenet Evol 2008 49 629 638 10.1016/j.ympev.2008.08.023 18804542

[B98] Gaither MR Toonen RJ Robertson DR Planes S Bowen BW Genetic evaluation of marine biogeographic barriers: perspectives from two widespread Indo-Pacific snappers (*Lutjanus *spp.) J Biogeogr 2010 37 133 147

[B99] Klanten OS Choat JH Van Herwerden L Extreme genetic diversity and temporal rather than spatial partitioning in a widely distributed coral reef fish Mar Biol 2007 150 659 670

[B100] Miller MP Bellinger RM Forsman ED Haig SM Effects of historical climate change, habitat connectivity and vicariance on the genetic structure and diversity across the range of the red tree vole (*Phenacomys longicaudus*) Mol Ecol 2006 15 145 159 16367837 10.1111/j.1365-294X.2005.02765.x

[B101] Mora MS Lessa EP Cutrera AP Kitttlein MJ Vasallo AI Phylogeographical structure in the subterranean tuco-tuco *Ctenomys talarum *(Rodentia: Ctenomyidea): constrasting the demographic consequences of regional and habitat specific histories Mol Ecol 2007 16 3453 3465 10.1111/j.1365-294X.2007.03398.x 17688545

[B102] Donaldson TJ Uyeno T, Arai R, Taniuchi T, Matsuura K Distribution and species richness patterns of Indo-West Pacific Cirrhitidae: support for Woodland's hypothesis Proceedings of the Second International Conference on Indo-Pacific fishes 1986 Tokyo: Ichthyological Society of Japan 623 628 1986; Tokyo

[B103] Hobbs JPA Frisch AJ Allen GR van Herwerden L Marine hybrid hotspot at Indo-Pacific biogeographic border Biology Letters 2009 5 258 261 19126528 10.1098/rsbl.2008.0561 PMC2665801

[B104] Marie AD van Herwerden L Choat JH Hybridization of reef fishes at the Indo-Pacific biogeographic barrier: a case study Coral Reefs 2007 26 841 850 10.1007/s00338-007-0273-3

[B105] Craig MT The goldrim surgeonfish (*Acanthurus nigricans*; Acanthuridae) from Diego Garcia, Chagos Archipelago: first record for the central Indian Ocean Zootaxa 2008 1850 65 68

[B106] Crandall ED Frey MA Grosberg RK Barber PH Contrasting demographic history and phylogeographical patterns in two Indo-Pacific gastropods Mol Ecol 2008 17 611 626 18179436 10.1111/j.1365-294X.2007.03600.x

[B107] Schultz JK Feldheim KA Gruber SH Ashley MV McGovern TM Bowen BW Global phylogeography and seascape genetics of the lemon sharks (genus *Negaprion*) Mol Ecol 2008 17 5336 5348 10.1111/j.1365-294X.2008.04000.x 19121001

[B108] Allen GR Smith-Vaniz WF Fishes of the Cocos (Keeling) Islands Atoll Res Bull 1994 412 1 21

[B109] Winterbottom R Anderson RC A revised checklist of the epipelagic and shore fishes of the Chagos Archipelago, Central Indian Ocean 1997 66 JLB Smith Institute of Ichthyology Ichthyological Bulletin 1 28

[B110] Briggs JC Bowen BW A realignment of marine biogeographic provinces with particular reference to fish distributions J Biogeogr 2011 in press

